# A Multicenter Randomized Controlled Trial To Evaluate the Efficacy and Safety of Nelfinavir in Patients with Mild COVID-19

**DOI:** 10.1128/spectrum.04311-22

**Published:** 2023-05-04

**Authors:** Taiga Miyazaki, Naoki Hosogaya, Yuri Fukushige, Sachiko Takemori, Shinpei Morimoto, Hiroshi Yamamoto, Makoto Hori, Yoshihito Ozawa, Yuki Shiko, Yosuke Inaba, Tomoya Kurokawa, Hideki Hanaoka, Shoya Iwanami, Kwangsu Kim, Shingo Iwami, Koichi Watashi, Ken Miyazawa, Takashi Umeyama, Satoshi Yamagoe, Yoshitsugu Miyazaki, Takaji Wakita, Makoto Sumiyoshi, Tatsuro Hirayama, Koichi Izumikawa, Katsunori Yanagihara, Hiroshi Mukae, Hitoshi Kawasuji, Yoshihiro Yamamoto, Norihito Tarumoto, Hiroshi Ishii, Hideaki Ohno, Kazuhiro Yatera, Hiroshi Kakeya, Yoshiko Kichikawa, Yasuyuki Kato, Tetsuya Matsumoto, Makoto Saito, Hiroshi Yotsuyanagi, Shigeru Kohno

**Affiliations:** a Division of Respirology, Rheumatology, Infectious Diseases, and Neurology, Department of Internal Medicine, Faculty of Medicine, University of Miyazaki, Miyazaki, Japan; b Nagasaki University, Nagasaki, Japan; c Clinical Research Center, Nagasaki University Hospital, Nagasaki, Japan; d Department of Respiratory Medicine, Nagasaki University Hospital, Nagasaki, Japan; e Division of Clinical Research Center, Chiba University Hospital, Chiba, Japan; f interdisciplinary Biology Laboratory (iBLab), Division of Biological Sciences, Graduate School of Science, Nagoya University, Aichi, Japan; g Research Center for Drug and Vaccine Development, National Institute of Infectious Diseases, Tokyo, Japan; h Department of Fungal Infection, National Institute of Infectious Diseases, Tokyo, Japan; i Section of Infectious Resource Coordination, Department of Research Resource, Center of Clinical Sciences, National Center for Global Health and Medicine, Tokyo, Japan; j National Institute of Infectious Diseases, Tokyo, Japan; k Department of Pharmacotherapeutics, Nagasaki University Graduate School of Biomedical Science, Nagasaki, Japan; l Infection Control and Education Center, Nagasaki University Hospital, Nagasaki, Japan; m Department of Laboratory Medicine, Nagasaki University Hospital, Nagasaki, Japan; n Department of Infectious Disease, Toyama University Hospital, Toyama, Japan; o Department of Infectious Disease and Infection Control, Saitama Medical University Hospital, Saitama, Japan; p Department of Respiratory Medicine, Fukuoka University Chikushi Hospital, Fukuoka, Japan; q Department of Infectious Disease and Infection Control, Saitama Medical Center, Saitama, Japan; r Department of Respiratory Medicine, Hospital of the University of Occupational and Environmental Health, Japan, Fukuoka, Japan; s Department of Infection Control Science, Osaka Metropolitan University Graduate School of Medicine, Osaka, Japan; t Division of Respiratory Medicine, Mishuku Hospital, Tokyo, Japan; u Department of Infectious Diseases, International University of Health and Welfare Narita Hospital, Chiba, Japan; v Department of Infectious Diseases and Applied Immunology, IMSUT Hospital of The Institute of Medical Science, The University of Tokyo, Tokyo, Japan; Cornell University College of Veterinary Medicine

**Keywords:** COVID-19, SARS-CoV-2, nelfinavir, protease inhibitor, randomized controlled trial

## Abstract

Nelfinavir, an orally administered inhibitor of human immunodeficiency virus protease, inhibits the replication of severe acute respiratory syndrome coronavirus 2 (SARS-CoV-2) *in vitro*. We conducted a randomized controlled trial to evaluate the clinical efficacy and safety of nelfinavir in patients with SARS-CoV-2 infection. We included unvaccinated asymptomatic or mildly symptomatic adult patients who tested positive for SARS-CoV-2 infection within 3 days before enrollment. The patients were randomly assigned (1:1) to receive oral nelfinavir (750 mg; thrice daily for 14 days) combined with standard-of-care or standard-of-care alone. The primary endpoint was the time to viral clearance, confirmed using quantitative reverse-transcription PCR by assessors blinded to the assigned treatment. A total of 123 patients (63 in the nelfinavir group and 60 in the control group) were included. The median time to viral clearance was 8.0 (95% confidence interval [CI], 7.0 to 12.0) days in the nelfinavir group and 8.0 (95% CI, 7.0 to 10.0) days in the control group, with no significant difference between the treatment groups (hazard ratio, 0.815; 95% CI, 0.563 to 1.182; *P *= 0.1870). Adverse events were reported in 47 (74.6%) and 20 (33.3%) patients in the nelfinavir and control groups, respectively. The most common adverse event in the nelfinavir group was diarrhea (49.2%). Nelfinavir did not reduce the time to viral clearance in this setting. Our findings indicate that nelfinavir should not be recommended in asymptomatic or mildly symptomatic patients infected with SARS-CoV-2. The study is registered with the Japan Registry of Clinical Trials (jRCT2071200023).

**IMPORTANCE** The anti-HIV drug nelfinavir suppresses the replication of severe acute respiratory syndrome coronavirus 2 (SARS-CoV-2) *in vitro*. However, its efficacy in patients with COVID-19 has not been studied. We conducted a multicenter, randomized controlled trial to evaluate the efficacy and safety of orally administered nelfinavir in patients with asymptomatic or mildly symptomatic COVID-19. Compared to standard-of-care alone, nelfinavir (750 mg, thrice daily) did not reduce the time to viral clearance, viral load, or the time to resolution of symptoms. More patients had adverse events in the nelfinavir group than in the control group (74.6% [47/63 patients] versus 33.3% [20/60 patients]). Our clinical study provides evidence that nelfinavir, despite its antiviral effects on SARS-CoV-2 *in vitro*, should not be recommended for the treatment of patients with COVID-19 having no or mild symptoms.

## INTRODUCTION

Severe acute respiratory syndrome coronavirus 2 (SARS-CoV-2), which has caused the coronavirus disease (COVID-19) pandemic, has been a global health concern since its identification in December 2019 (1). Although vaccination is the mainstay of management for this pandemic, the emergence of new viral variants may diminish its efficacy. Therefore, effective treatments against SARS-CoV-2 are needed. Currently, remdesivir and immunosuppressive/anti-inflammatory drugs (e.g., dexamethasone) are used to treat hospitalized patients with moderate to severe COVID-19 (2). Two oral antiviral agents, ritonavir-boosted nirmatrelvir and molnupiravir, have recently received emergency use authorization from the Food and Drug Administration for the treatment of patients with mild COVID-19 who are at a high risk of disease progression (3, 4). Because transmission frequently occurs even from asymptomatic patients, developing new oral antiviral agents for the treatment of patients with asymptomatic or mildly symptomatic COVID-19 is crucial (5).

Nelfinavir is an orally administered inhibitor of human immunodeficiency virus protease (6, 7). In a nonclinical study using a cell culture model of SARS-CoV-2 infection, nelfinavir suppressed viral replication by inhibiting the main protease (8). However, its efficacy in patients with SARS-CoV-2 infection has not been studied. In previous randomized clinical trials, lopinavir-ritonavir and remdesivir did not show significant benefits for patients with severe COVID-19 (9, 10); however, these findings may have been observed because the treatments were initiated too late: after 13 and 11 days since the onset of symptoms, respectively. The mathematical model for virus dynamics also suggests that for clinical trials with a small sample size investigating the efficacy of antiviral drugs, patients should be recruited as early as possible after symptom onset to observe statistically significant results (11). On the basis of these findings, we designed and conducted an exploratory, randomized, controlled trial to evaluate the clinical antiviral efficacy and safety of nelfinavir in patients with asymptomatic and mildly symptomatic COVID-19.

## RESULTS

**FIG 1:**
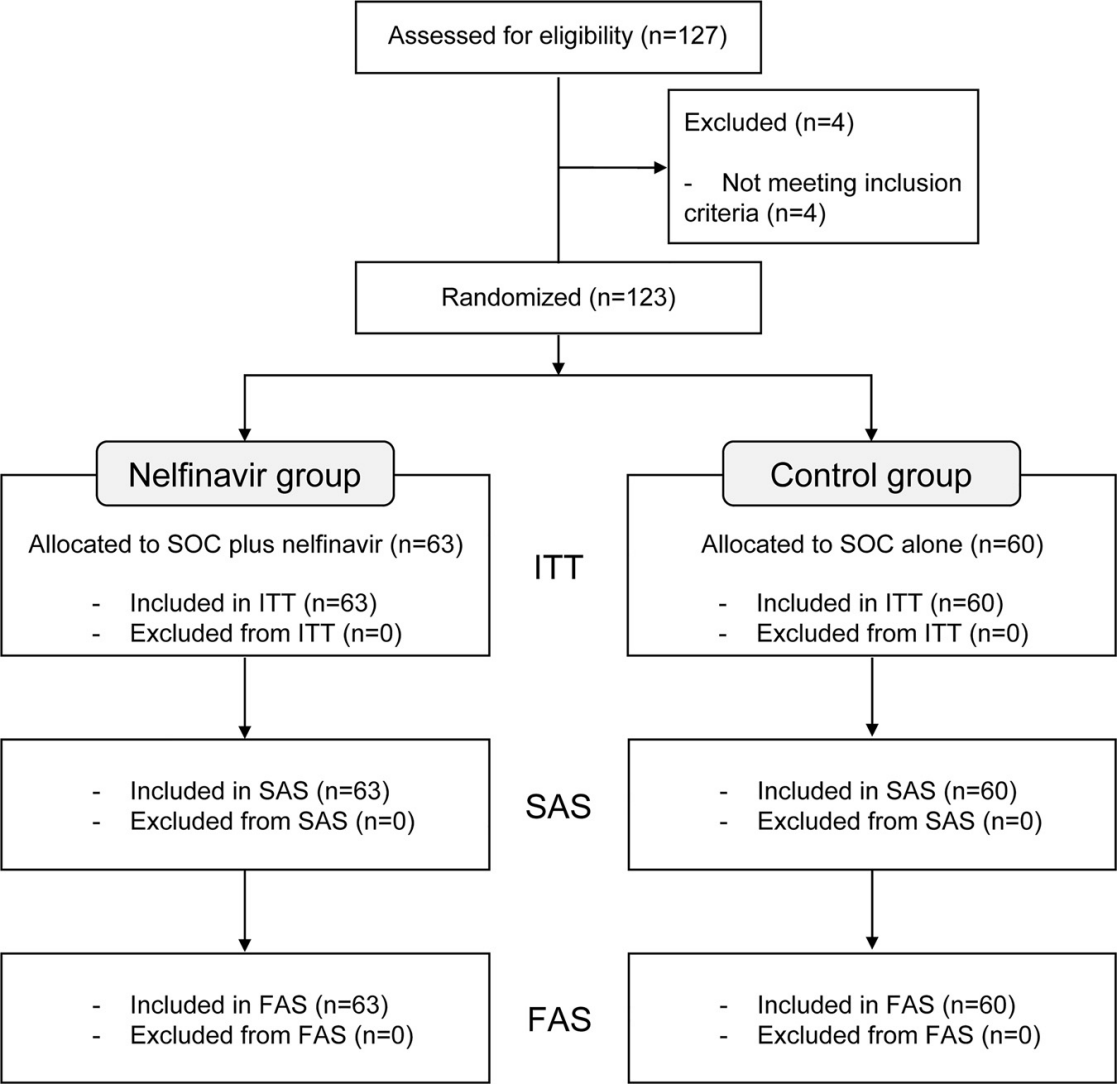
Flowchart of the study patients. Analysis sets: intention-to-treat (ITT) population, all randomly assigned patients; safety analysis set (SAS) and full analysis set (FAS), all randomly assigned patients who received the study treatment at least once. SOC, standard-of-care.

During the study, 127 patients were assessed for eligibility, and 123 were randomly assigned to the study arms: 63 in the nelfinavir group and 60 in the control group (Fig. 1). All patients included in the analysis received standard-of-care. Of these 123 patients, 116 completed the study: 59 (93.7%) and 57 (95.0%) in the nelfinavir and control groups, respectively. The other seven patients (four in the nelfinavir group and three in the control group) discontinued the study because of disease progression (three in the nelfinavir group and one in the control group), withdrawal of consent (one in each group), and protocol deviation (one in the control group).

Patients’ baseline characteristics are summarized in Table 1. The mean ± SD age was 42.7 ± 13.5 and 47.8 ± 15.4 years in the nelfinavir and control groups, respectively. Further, 41 (65.1%) and 33 (55.0%) patients in the nelfinavir and control groups, respectively, were males. Most patients had one or more mild symptoms; 6 (9.5%) and 5 (8.3%) patients in the nelfinavir and control groups, respectively, were asymptomatic. Proportions of the Alpha and Delta variants were similar between the two groups.

**TABLE 1:** Baseline characteristics of the study patients^*a*^

Characteristic	Nelfinavir group (*n* = 63)	Control group (*n* = 60)
Sex, *n* (%)		
Female	22 (34.9)	27 (45.0)
Male	41 (65.1)	33 (55.0)
Age (years)		
Mean (SD)	42.7 (13.5)	47.8 (15.4)
Range	20.0 to 75.0	20.0 to 89.0
Age, *n* (%)		
<60 years	53 (84.1)	49 (81.7)
≥60 years	10 (15.9)	11 (18.3)
Severity, *n* (%)		
Asymptomatic	6 (9.5)	5 (8.3)
Mild	57 (90.5)	55 (91.7)
History/complication, *n* (%)		
No	27 (42.9)	23 (38.3)
Yes	36 (57.1)	37 (61.7)
Concomitant drugs/therapies, *n* (%)		
No	4 (6.3)	9 (15.3)
Yes	59 (93.7)	50 (84.7)
Missing^*b*^	0	1
SARS-CoV-2 test method/positive for COVID-19, *n* (%)		
PCR^*c*^	60 (95.2)	50 (83.3)
LAMP	1 (1.6)	7 (11.7)
Antigen test	2 (3.2)	3 (5.0)
Morbidity duration (days), *n* (%)		
0	5 (7.9)	6 (10.0)
1	4 (6.3)	7 (11.7)
2	15 (23.8)	19 (31.7)
3	14 (22.2)	17 (28.3)
4	11 (17.5)	4 (6.7)
5	8 (12.7)	6 (10.0)
6	5 (7.9)	1 (1.7)
7	1 (1.6)	0 (0.0)
Morbidity duration (days)		
Mean (SD)	3.1 (1.7)	2.5 (1.4)
Range	0.0 to 7.0	0.0 to 6.0
Day 1 viral load (× 10^3^ per 17 μL)^*d*^		
Mean (SD)	2,225 (12,039)	614 (4,220)
Range	0.0 to 93,700	0.0 to 32,700
Viral genome sequencing, *n* (%)		
Nextclade (iVar)^*e*^	*n* = 38	*n* = 28
20B + 20C	17 (44.7)	13 (46.4)
20I (Alpha)	10 (26.3)	6 (21.4)
21A (Delta)	8 (21.1)	5 (17.9)
Sequencing failure	3 (7.9)	4 (14.3)

a SD, standard deviation; LAMP, loop-mediated isothermal amplification.

b Patient who discontinued.

c Including 11 (nelfinavir group) and 6 (control group) tested for both PCR and antigen test.

d Viral load was measured using only quantitative RT-PCR.

e Nextclade and corresponding Pangolin lineage were as follows: 20B: B.1.1, B.1.1.214, B.1.1.284, and R.1; 20C: B.1.346; 20I (Alpha): B.1.1.7; 21A (Delta): B.1.617.2 and AY.29.

**FIG 2:**
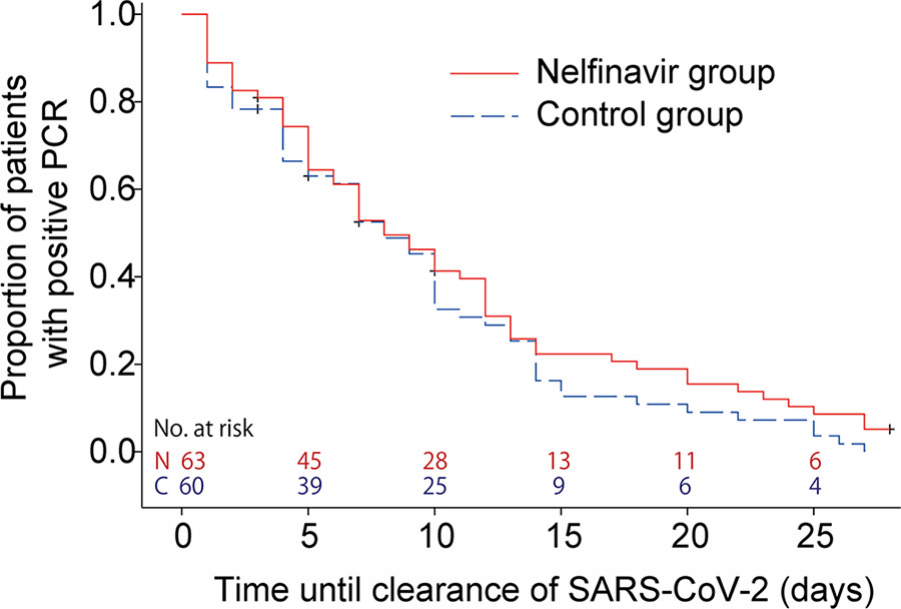
Time to clearance of severe acute respiratory syndrome coronavirus 2 (SARS-CoV-2). The median time to viral clearance from randomization is 8.0 (95% confidence interval [CI], 7.0 to 12.0) days in the nelfinavir group and 8.0 (95% CI, 7.0 to 10.0) days in the control group (*P *= 0.1870, stratified log-rank test adjusted for severity and age). Hazard ratio of the viral clearance for the nelfinavir group versus the control group was 0.815 (95% CI, 0.563 to 1.182; Cox regression analysis adjusted for severity and age).

**FIG 3:**
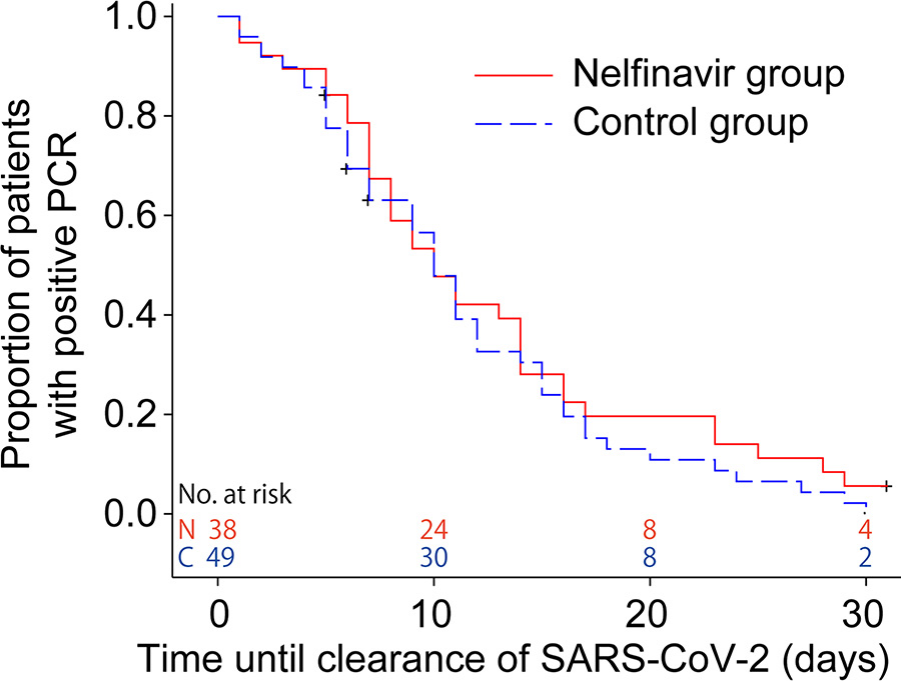
Subgroup analysis of the time to clearance of severe acute respiratory syndrome coronavirus 2 (SARS-CoV-2) from symptom onset in patients treated within 3 days of symptom onset. The median time to viral clearance is 13.0 (95% confidence interval [CI], 10.0 to 17.0) days in the nelfinavir group and 13.0 (95% CI, 10.0 to 14.0) days in the control group (*P *= 0.2312, stratified log-rank test adjusted for severity and age).

After initiating the study, 57 patients in each group had two consecutive negative RT-PCR results. The median time to viral clearance was 8.0 (95% confidence interval [CI], 7.0 to 12.0) days in the nelfinavir group and 8.0 (95% CI, 7.0 to 10.0) days in the control group (Fig. 2). The hazard ratio of the viral clearance for the nelfinavir group versus the control group was 0.815 (95% CI, 0.563 to 1.182). The intergroup difference was not significant (*P *= 0.1870). This study enrolled patients who tested positive for SARS-CoV-2 infection within 3 days of enrollment. The patients were asymptomatic or within 7 days of symptom onset. Among them, a subgroup analysis of patients whose treatment was initiated within 3 days of symptom onset (*n* = 38 in the nelfinavir group and *n* = 49 in the control group; Table 1) also showed similar results (Fig. 3); the median time from symptom onset to viral clearance was 13.0 (95% CI, 10.0 to 17.0) days in the nelfinavir group and 13.0 (95% CI, 10.0 to 14.0) days in the control group (*P *= 0.2312).

**FIG 4:**
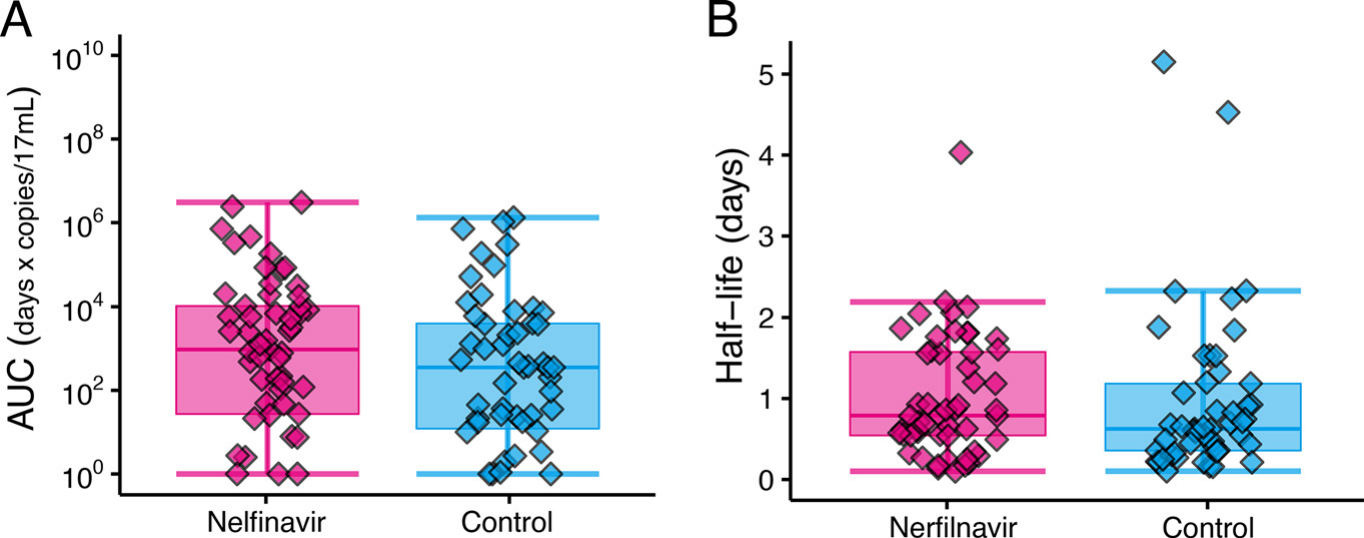
Distributions of area under the curve (AUC) and half-life of viral load. Individual values of (A) AUC and (B) half-life of viral load for patients in the nelfinavir and control groups with box plots that show the quantiles. AUC was calculated as the summation of the product of the measured viral load and the measurement interval. Half-life was estimated using only measurements above the detection limit after attaining the peak of viral load.

When comparing only the viral load on day 1 (Table 1), a large apparent difference with substantial SDs was introduced by chance in the randomized allocation. However, the difference at this single time point may not necessarily have a substantial impact on protocol outcomes as viruses increase exponentially. The efficacy of treatment should be evaluated based on viral kinetics and infection dynamics. The individual viral loads over time for each patient and time course changes in viral load for all patients in each group are shown in Fig. S1 and S2 in the supplemental material, respectively. There was no significant difference in the area under the curve (AUC) and half-life of the viral load between the two groups (Fig. 4 and Table S1).

During the study, COVID-19-related symptoms resolved in most patients within 28 days in both groups, but dysgeusia and anosmia persisted in 15 to 30% of the patients (Table 2). The median time to resolution of all symptoms was 17 (95% CI, 14 to 23) days in the nelfinavir group and 18 (95% CI, 13 to 26) days in the control group (*P *= 0.8101). The median time to resolution of each symptom was also similar between the treatment groups.

**TABLE 2:** Resolution of symptoms in patients with mild COVID-19^*a*^

	Nelfinavir group (*n* = 57)			Control group (*n* = 55)			
Symptom	No. of patients with a symptom (%)	Percentage of patients in whom the symptom resolved (%)^*b*^	Median days to resolution (95% CI)	No. of patients with a symptom (%)	Percentage of patients in whom the symptom resolved (%)^*b*^	Median days to resolution (95% CI)	*P* value^*c*^
Fever	47 (82.5)	93.6 (44/47)	2 (1–3)	49 (89.1)	98.0 (48/49)	3 (2–4)	0.4324
All symptoms	57 (100.0)	63.2 (36/57)	17 (14–23)	55 (100.0)	60.0 (33/55)	18 (13–26)	0.8101
Cough	51 (89.5)	92.2 (47/51)	7 (4–10)	48 (87.3)	89.6 (43/48)	8 (4–11)	0.8421
Dyspnea	21 (36.8)	85.7 (18/21)	2 (1–3)	18 (32.7)	83.3 (15/18)	4.5 (1–12)	0.1777
Sputum	41 (71.9)	82.9 (34/41)	8 (3–10)	43 (78.2)	90.7 (39/43)	5 (2–8)	0.3283
Nasal discharge	32 (56.1)	90.6 (29/32)	5 (2–7)	31 (56.4)	87.1 (27/31)	3 (2–4)	0.7042
Nasal congestion	32 (56.1)	93.8 (30/32)	5 (3–8)	29 (52.7)	86.2 (25/29)	5 (2–6)	0.8025
Pharyngeal pain	37 (64.9)	94.6 (35/37)	3 (2–6)	33 (60.0)	97.0 (32/33)	3 (2–5)	0.7027
Malaise	48 (84.2)	89.6 (43/48)	4.5 (2–6)	42 (76.4)	95.2 (40/42)	5 (3–8)	0.7427
Headache	35 (61.4)	94.3 (33/35)	3 (2–7)	40 (72.7)	95.0 (38/40)	4 (2–5)	0.5975
Decreased appetite	37 (64.9)	94.6 (35/37)	4 (2–6)	34 (61.8)	100.0 (34/34)	2 (1–4)	0.1199
Diarrhea	47 (82.5)	95.7 (45/47)	2 (1–5)	30 (54.5)	96.7 (29/30)	2 (1–3)	0.1156
Nausea	16 (28.1)	87.5 (14/16)	1.5 (1–4)	15 (27.3)	100.0 (15/15)	1 (1–2)	0.7495
Vomiting	6 (10.5)	100.0 (6/6)	1 (1–3)	6 (10.9)	100.0 (6/6)	1 (1–5)	0.5514
Arthralgia	25 (43.9)	100.0 (25/25)	2 (1–6)	22 (40.0)	100.0 (22/22)	4 (2–6)	0.3209
Myalgia	22 (38.6)	100.0 (22/22)	3 (2–5)	22 (40.0)	100.0 (22/22)	3 (1–4)	0.8572
Dysgeusia	24 (42.1)	82.6 (19/24)	9 (4–15)	20 (36.4)	85.0 (17/20)	5 (2–11)	0.5403
Anosmia	29 (50.9)	72.4 (21/29)	11 (4–19)	20 (36.4)	70.0 (14/20)	19 (4–∞)	0.5487

a CI, confidence interval.

b Per no. of patients with the symptom.

c log-rank test.

During the study, 47 (74.6%) patients in the nelfinavir group and 20 (33.3%) in the control group experienced at least one adverse event (Table 3). No patients died because of such adverse events. The most common adverse events were diarrhea (49.2%) in the nelfinavir group and rash (6.7%) in the control group. Serious adverse events (SAEs), which were bradycardia, diarrhea, and hepatic dysfunction, occurred in three patients in the nelfinavir group. Of these, bradycardia was not considered by the investigator to be related to the study treatment. All SAEs led to treatment discontinuation. Of these, bradycardia and diarrhea resolved after treatment discontinuation, whereas hepatic dysfunction did not resolve until the end of follow-up.

**TABLE 3:** Adverse events occurring in at least two patients in any group

		Nelfinavir group (*n* = 63)		Control group (*n* = 60)	
System organ class	Preferred term	No. of patients (%)	No. of events	No. of patients (%)	No. of events
Total		47 (74.6)	91	20 (33.3)	46
Gastrointestinal disorders		37 (58.7)	44	7 (11.7)	11
	Constipation	1 (1.6)	1	2 (3.3)	2
	Diarrhea	31 (49.2)	31	3 (5.0)	3
	Nausea	4 (6.3)	4	1 (1.7)	1
	Vomiting	2 (3.2)	2	0 (0.0)	0
General disorders and administration site conditions		4 (6.3)	4	1 (1.7)	1
	Pyrexia	2 (3.2)	2	1 (1.7)	1
Investigations		5 (7.9)	6	3 (5.0)	5
	Neutrophil count decreased	2 (3.2)	2	0 (0.0)	0
Nervous system disorders		5 (7.9)	6	4 (6.7)	4
	Hypoesthesia	1 (1.6)	1	2 (3.3)	2
Respiratory, thoracic, and mediastinal disorders		2 (3.2)	2	4 (6.7)	4
	Dysphonia	0 (0.0)	0	2 (3.3)	2
Skin and subcutaneous tissue disorders		17 (27.0)	18	5 (8.3)	5
	Drug eruption	3 (4.8)	3	0 (0.0)	0
	Pruritus	2 (3.2)	2	0 (0.0)	0
	Rash	11 (17.5)	11	4 (6.7)	4

The concomitant drugs used during the study period (days 1 to 28) are listed in Table S2. More patients took antidiarrheals in the nelfinavir group than in the control group (33.3% [21/63] versus 6.7% [4/60]), given the increased frequency of diarrhea as an adverse event of nelfinavir. The numbers of patients who took certain types of drugs, including steroids, antipyretics, antitussives, and antivirals, that may affect symptoms, morbidity, severity, or time for viral clearance were similar between the two groups.

## DISCUSSION

In this exploratory, randomized, controlled clinical trial, compared with standard-of-care alone, nelfinavir combined with standard-of-care treatment did not significantly reduce the time to clearance of SARS-CoV-2. Analyses of the secondary endpoints, viral load, and COVID-19-related symptoms indicated similar results. These results do not support the use of nelfinavir for the treatment of patients infected with SARS-CoV-2 having no or mild COVID-19 symptoms.

In this study, the dosage of nelfinavir (750 mg; thrice daily) was based on the following reports. First, a mathematical model simulating the treatment of SARS-CoV-2 infection with nelfinavir suggested that a dosage of 500 mg twice daily was sufficient to reduce viral load (8). This model combined pharmacodynamic parameters measured in cultured cells with the following findings: (i) pharmacokinetic parameters in the lung estimated from the results of previous preclinical and clinical studies (12 to 14), and (ii) viral infection dynamics estimated from the time course data on viral shedding after symptom onset in patients with COVID-19 (15). In this mathematical model simulation, 500 mg nelfinavir (twice daily) was the minimum dose, whereby pharmacokinetic parameters could be obtained from the previous studies (12 to 14), which would provide an adequate therapeutic effect on the reduction of the time to clearance. Second, 750 mg of nelfinavir (thrice daily) was the approved dosage for the treatment of HIV infections in Japan. This dosage is also approved in the United States (16), and the safety profile of this dosage is well established. Higher drug concentration and drug efficacy than that of 500 mg twice daily suggested by the mathematical model are expected to be achieved with a dosage of 750 mg thrice daily. The other approved dosage, 1,250 mg twice daily, was not selected in this study because of the small number of subjects expected to be enrolled.

However, it is unclear whether the dosage is adequate to suppress viral replication in the respiratory epithelial cells and lung tissues of patients with COVID-19. Although the results of a previous study showed that after oral administration, the average concentration of nelfinavir was 3.24 times higher in the lung than in the plasma of rats (13), limited data are available regarding its distribution in human tissues. Therefore, the dosage of nelfinavir used in the present study might not have resulted in a sufficient concentration of the drug in the lung tissues to exert potent activity against SARS-CoV-2. In addition, high human plasma protein binding might reduce the antiviral activity of nelfinavir to some extent (17).

Another possible explanation for the lack of potent activity is the emergence of the B.1.617.2 (Delta) variant of SARS-CoV-2. When Ohashi et al. reported that nelfinavir inhibited the catalytic activity of the SARS-CoV-2 main protease in a dose-dependent manner (8), they used the Wk-521 strain isolated from a patient with COVID-19 (18). Conversely, when this study was conducted, the Alpha and Delta variants began to spread across Japan, which might have diminished the effect of nelfinavir. Approximately half of the SARS-CoV-2 strains sequenced in this study were variants of concern, including the Alpha and Delta variants in both groups. However, the main protease of the Alpha and Delta variants are identical to that of the wild type (19). Moreover, PF-07304814 and nirmatrelvir, inhibitors of the main protease of SARS-CoV-2, were effective even against the B.1.1.529 (Omicron) variant (20, 21), which has an amino acid change in the main protease (21). Therefore, this explanation may not be valid.

The *post hoc* subgroup analysis conducted in the present study revealed that the time to viral clearance was similar between the treatment groups, even after the exclusion of patients treated after the third day from symptom onset. This result does not mean that nelfinavir does not affect viral replication, because treatments may sometimes induce a longer duration of viral shedding. Compared with no treatment, early initiation of treatments with intermediate to high efficacy prolongs the duration of viral shedding, because progeny viruses that escape from treatment further infect previously uninfected cells at a lower rate (22). Furthermore, in a recent report on the SARS-CoV-2 human challenge, no clear antiviral effects on the duration of viral shedding were found even when preemptive remdesivir treatment was started immediately after two consecutive positive RT-PCR results at a 12-h interval (23).

The most frequent adverse events in patients receiving nelfinavir were diarrhea and nausea. These adverse events were also reported in the clinical trials of patients infected with HIV, wherein diarrhea was the most frequently reported adverse event (6, 16). However, diarrhea is considered to be generally mild and usually controlled with drugs that slow gastrointestinal motility (6). In the present study, severe diarrhea resolved with treatment discontinuation.

Some limitations of the present study should be mentioned. First, the sample size was not sufficiently large to assess the time to disease progression. Therefore, this outcome was not incorporated into the present exploratory study. Although preventing disease progression is the primary objective of treating patients infected with SARS-CoV-2, a large-scale, randomized, controlled trial is needed to assess the effect of nelfinavir treatment on this outcome. Second, the concentration of nelfinavir in the lung was not measured. Third, bias in assessing the symptoms of patients could not be minimized, as an open-label scheme was used.

In conclusion, nelfinavir combined with standard-of-care was not associated with a reduction in the time to viral clearance in patients infected with SARS-CoV-2 who had no or mild symptoms. During the course of this clinical trial, the findings of three experimental studies of nelfinavir treatment using the Syrian hamster model of SARS-CoV-2 infection were reported. First, Jan et al. showed that nelfinavir reduced the viral titer in the lung of SARS-CoV-2-infected Syrian hamsters (24). Bakowski et al. demonstrated that nelfinavir suppressed the viral replication *in vitro* but failed to demonstrate any antiviral effects *in vivo* (25). Finally, Foo et al. revealed that nelfinavir improved pulmonary pathology in SARS-CoV-2-infected Syrian hamsters but did not reduce the viral load in the lungs (26). Taken together, the major limitations of nelfinavir as a potential therapeutic agent for COVID-19 are its insufficient antiviral activity and the frequent occurrence of adverse events, including SAEs. Although several drugs are available for the treatment of COVID-19, no treatments are recommended for asymptomatic patients. Therefore, a new agent needs to be developed for such patients.

## MATERIALS AND METHODS

### Study design and ethical considerations.

This was a prospective, randomized, open-label, blinded-endpoint, parallel-group trial conducted between July 2020 and October 2021 at 11 universities and teaching hospitals in Japan. The study was conducted in accordance with the Declaration of Helsinki and compliance with Good Clinical Practice and other applicable regulatory requirements. Its protocol was approved by the institutional review board of Nagasaki University Hospital (approval number: I20-001) and the local review board of each participating institution. All patients provided written informed consent. The study was registered with the Japan Registry of Clinical Trials (jRCT2071200023). This report follows the CONSORT guidelines (27).

### Study population.

The study design has been previously reported (28). Briefly, Japanese adult patients (minimum 20 years old) were eligible for the study if they tested positive for SARS-CoV-2 infection within 3 days before enrollment. Infection was confirmed using reverse transcription-PCR (RT-PCR), loop-mediated isothermal amplification performed on a respiratory tract specimen (e.g., nasopharyngeal swab or saliva), or an antigen test. Patients had to be asymptomatic or have one or more mild symptoms of COVID-19, such as fever, cough, or dyspnea, without needing oxygen support.

Patients were excluded if they had a history of SARS-CoV-2 vaccination or if they wished to receive vaccination during the study period. Other exclusion criteria were as follows: (i) onset of symptoms ≥8 days before enrollment; (ii) oxygen saturation measured by pulse oximetry <96% on room air; (iii) inadequate hepatic or renal function ([i] alanine aminotransferase or aspartate aminotransferase ≥5 × upper limit of the reference range, [ii] Child–Pugh class B or C, or [iii] serum creatinine ≥2 × upper limit of the reference range and creatinine clearance <30 mL/min); (iv) poorly controlled diabetes mellitus (random blood glucose ≥200 mg/dL or hemoglobin A1c ≥7.0% despite treatment); or (v) severe diarrhea. Pregnant or breastfeeding women, women who might become pregnant during the study, or patients who did not use adequate contraceptive precautions were also excluded.

### Study arms.

This study consisted of a 14-day treatment period and a 14-day follow-up period. Before starting the treatment period, patients were randomly assigned in a 1:1 ratio to receive nelfinavir combined with standard-of-care, or standard-of-care alone. Random and concealed allocation was implemented using an interactive web response system with the dynamic allocation method adjusted for severity (asymptomatic versus mild symptom) and age (20 to 59 versus ≥60 years).

Nelfinavir (750 mg; thrice daily) was administered orally for 14 days. However, the treatment was to be discontinued based on the decision of the investigator if a patient had two consecutive negative RT-PCR results. Nelfinavir was supplied by Japan Tobacco, Inc. (Tokyo, Japan) and Pfizer, Inc. (New York, NY).

### Outcomes.

From the beginning of the treatment (day 1) to the end of the follow-up period (day 28), saliva samples were obtained from patients once daily, and viral load was measured only using quantitative RT-PCR at the National Institute of Infectious Diseases (Tokyo, Japan). Furthermore, patients kept a daily diary to record the presence or absence of COVID-19-related symptoms, such as cough, dyspnea, or headache, from day 1 to day 28. They also recorded any untoward symptom as an adverse event. Abnormal findings in vital signs or laboratory values were collected if the investigators considered them necessary. Viral load data obtained before enrollment were not used for the analyses.

The primary endpoint was the time to clearance of SARS-CoV-2, which was adjudicated by the assessors at the central laboratory. The assessors were blinded to the assigned treatment. Viral clearance was defined as negative results for two consecutive RT-PCR assays conducted using saliva samples. Time to viral clearance was defined as the time from the inclusion to the day when the first negative result was obtained in case of viral clearance.

The secondary endpoints included the AUC and the half-life of the viral load. In addition to these secondary endpoints, the time to resolution of all COVID-19-related symptoms and that of each symptom were recorded for patients with mild symptoms. An Effectiveness and Safety Assessment Committee was established independently of the physicians associated with the trial to review the overall safety and efficacy of the trial following standard operating procedures.

### Viral genome sequencing.

RNA was extracted from the saliva samples exhibiting Cp of <31 in quantitative RT-PCR of SARS-CoV-2. cDNA synthesis, multiplex PCR, and Illumina library prep (Illumina K.K., Tokyo, Japan) were performed as described previously (29). The samples were purified using AMPureXP (Beckman Coulter, Tokyo, Japan) and sequenced for 151 cycles, and paired-end reads were generated on Illumina iSeq100 (Illumina). Sequence analyses were performed using the nf-core/viralrecon pipeline (30).

### Statistical analysis.

Details of sample size calculation were explained in the previous article (28). In brief, a sample size of 60 patients in each group was calculated to be sufficient to detect the differences in the time to the viral clearance between the treatment groups, with an assumed hazard ratio of 1.79 and a power of 80%. This assumption was obtained from a simulation of viral dynamics.

Baseline characteristics were expressed as mean ± standard deviation (SD) or number with a percentage. Following the CONSORT guidelines (27), we did not perform significance tests of baseline differences that could have occurred by chance in the random allocation. Efficacy outcomes were analyzed using the intention-to-treat population, which consisted of all randomly assigned patients. Safety outcomes were analyzed through the descriptive summary of adverse reactions for each treatment group using the safety analysis set. Patients who received at least one dose of nelfinavir were assigned to the nelfinavir group.

We estimated the time to viral clearance using the Kaplan-Meier survival curves for each treatment group. For intergroup comparison of survival data, we used the log-rank test to detect the effect of nelfinavir. The hazard ratio and its 95% CI were calculated using a Cox proportional hazards model. Regarding the secondary endpoints, the cumulative viral load displayed as AUC was calculated as the sum of the product of the measured viral load and the measurement interval. The difference in AUC of viral load between the nelfinavir and control groups was evaluated using an equal variance two-sample *t* test. The half-life of the viral load was estimated for each patient using only measurements above the detection limit after attaining the peak of the viral load. The time to resolution of all symptoms or each symptom was analyzed as time-to-event data, and intergroup comparison was performed using the log-rank test. A *post hoc* subgroup analysis was conducted in patients who were treated within 3 days of symptom onset. All data were analyzed using SAS software version 9.4 (SAS institute, Cary, NC). All reported *P* values are two-tailed.

### Data availability.

The data that support the findings of this study are available from the corresponding author upon special request.
