# Supplementary material for: A Multicenter Randomized Controlled Trial To Evaluate the Efficacy and Safety of Nelfinavir in Patients with Mild COVID-19
Source: Microbiol Spectr. 2023 May 4;11(3):e04311-22. doi: 10.1128/spectrum.04311-22 (PMC10269734; doi:10.1128/spectrum.04311-22)
Supplement: Supplemental file 1 — Supplemental material. Download spectrum.04311-22-s0001.pdf, PDF file, 0.8 MB [file spectrum.04311-22-s0001.pdf]

A multicenter randomized controlled trial to evaluate the efficacy and safety of nelfinavir in patients with mild COVID-19

Miyazaki T, et al.

Supplemental Material

Supplementary Figure S1

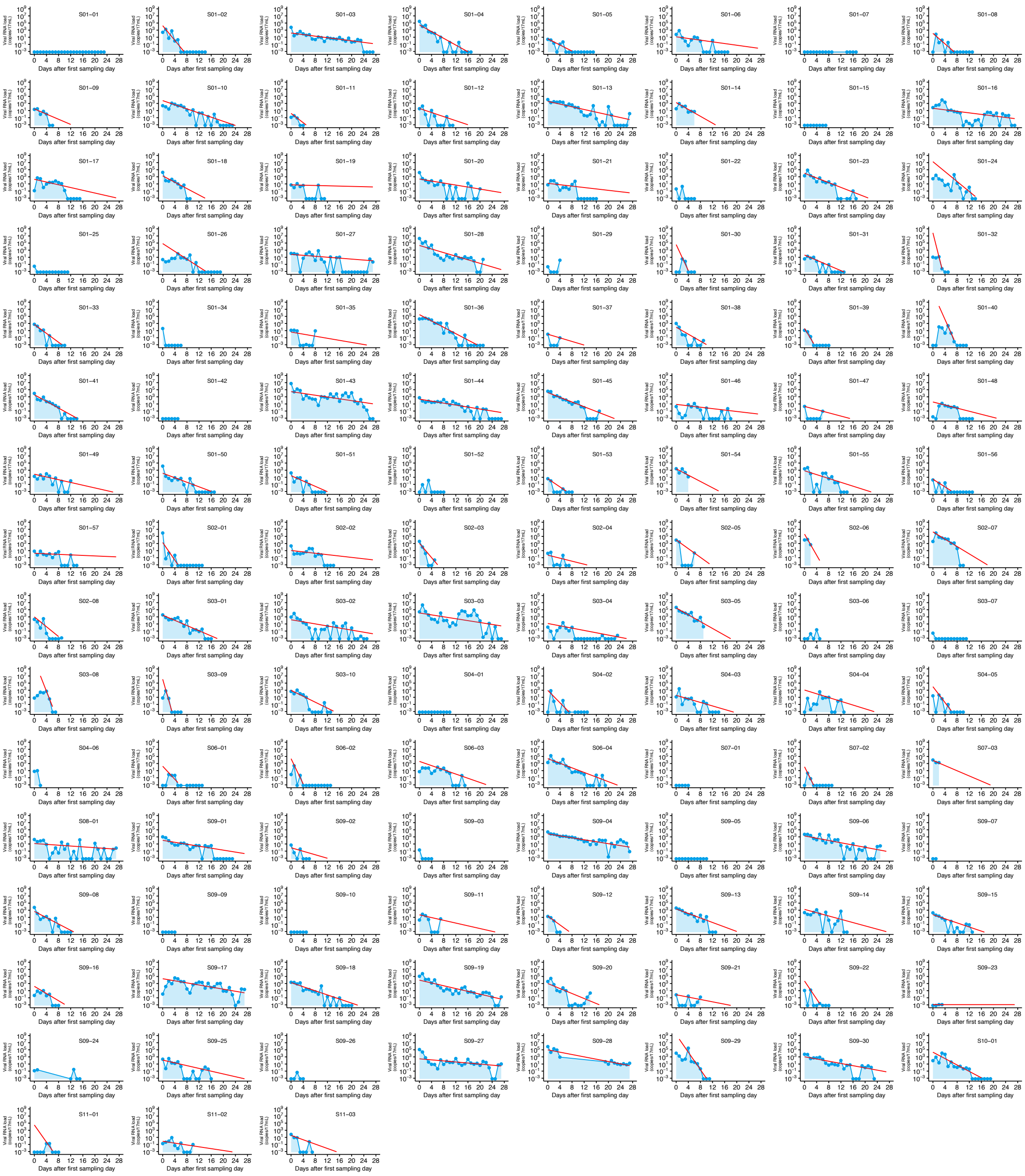

Supplementary Figure S1. Individual viral loads over time

The values of individual viral loads over time for each patient (dots) are shown along with AUC (filled areas) and estimated slope of viral clearance (lines). The measurements under the detection limit were plotted as 10^{-3} copies/17mL.

Supplementary Figure S2

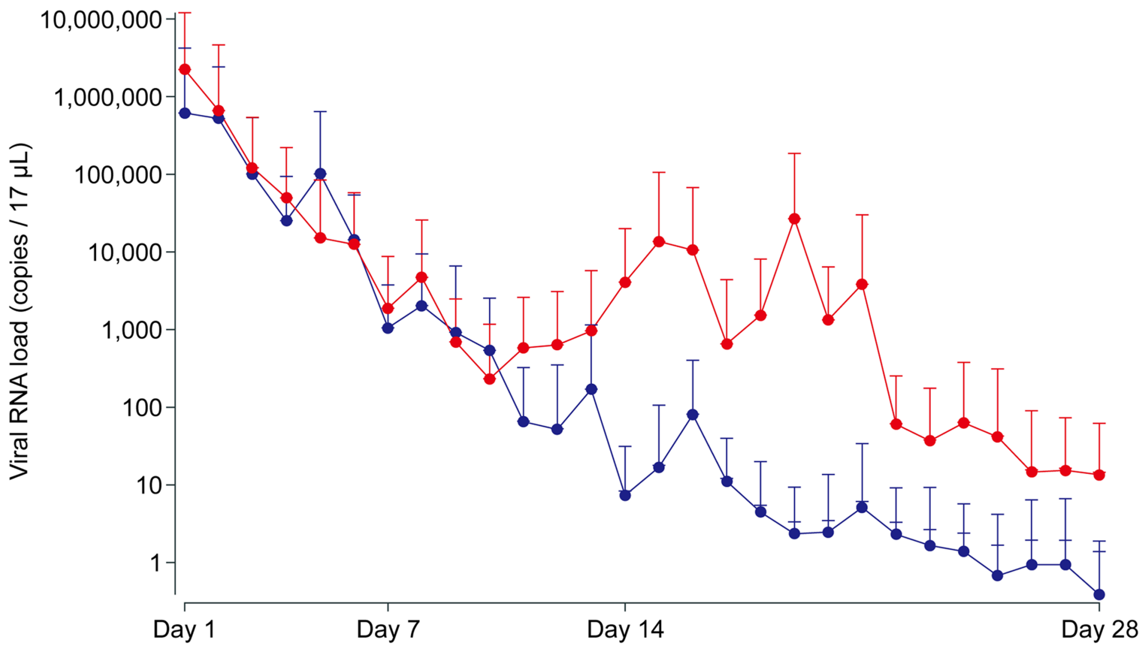

Supplementary Figure S2. Time-course changes in viral loads for all patients in each group

The line chart shows the average viral load over time. Error bars indicate only the upper end of the standard deviation.

Supplementary Table S1. Viral Dynamics: AUC and Half-life of Viral Load

	Nelfinavir group (N = 63)	Control group (N = 60)	*P*-value*
**$AUC_{(0\text{-}T)}$ (days × copies/17 mL)**			
N (missing)	62 (1)	60 (0)	0.4049
Mean (SD)	122,329.8 (496,818.3)	63,229.4 (234,228.7)	
Range	0.0–3,052,538.5	0.0–1,316,333.5	
**$T_{1/2}$ (days)**			
N (missing)	52 (11)	49 (11)	0.7023
Mean (SD)	1.0 (0.8)	1.0 (1.0)	
Range	0.1–4.0	0.1–5.1	

* Equal variance two sample *t*-test

AUC, area under the curve; SD, standard deviation

Supplementary Table S2. Concomitant drugs used during the study period

Classification / Ingredient	Nelfinavir group (N = 63)	Control group (N = 60)
Total, n (%)	56 (88.9)	45 (75.0)
Anticoagulants		
Heparin calcium	2 (3.2)	3 (5.0)
Tranexamic Acid	1 (1.6)	2 (3.3)
Sodium bicarbonate	1 (1.6)	0 (0.0)
Edoxaban tosilate hydrate	0 (0.0)	1 (1.7)
Ascorbic Acid	0 (0.0)	1 (1.7)
Mecobalamin	0 (0.0)	1 (1.7)
Antimicrobial agents		
Remdesivir	8 (12.7)	8 (13.3)
Casirivimab (genetical recombination) and imdevimab (genetical recombination)	0 (0.0)	1 (1.7)
Amoxicillin hydrate and potassium clavulanate	1 (1.6)	0 (0.0)
Ampicillin sodium and sulbactam sodium	1 (1.6)	0 (0.0)
Clarithromycin	1 (1.6)	0 (0.0)
Garenoxacin mesilate hydrate	1 (1.6)	0 (0.0)
Minocycline hydrochloride	0 (0.0)	1 (1.7)
Antipyretics and common cold drugs		
Acetaminophen	36 (57.1)	34 (56.7)
Salicylamide, acetaminophen, anhydrous caffeine, and promethazine methylenedisalicylate	2 (3.2)	0 (0.0)
Loxoprofen sodium hydrate	13 (20.6)	12 (20.0)
Celecoxib	1 (1.6)	2 (3.3)
Ibuprofen	1 (1.6)	0 (0.0)
Diclofenac sodium	0 (0.0)	1 (1.7)
Digestive organ agents		
Loperamide hydrochloride	21 (33.3)	4 (6.7)
Rebamipide	9 (14.3)	4 (6.7)

Vonoprazan fumarate	3 (4.8)	2 (3.3)
Lansoprazole	1 (1.6)	4 (6.7)
Esomeprazole magnesium hydrate	1 (1.6)	1 (1.7)
Rabeprazole sodium	1 (1.6)	1 (1.7)
Famotidine	1 (1.6)	0 (0.0)
Teprenone	1 (1.6)	0 (0.0)
Sennosides	2 (3.2)	4 (6.7)
Magnesium oxide	2 (3.2)	2 (3.3)
Precipitated calcium carbonate	1 (1.6)	0 (0.0)
Sodium bicarbonate and anhydrous monobasic sodium phosphate	1 (1.6)	0 (0.0)
Clostridium butyricum	8 (12.7)	6 (10.0)
Metoclopramide	3 (4.8)	5 (8.3)
Domperidone	0 (0.0)	1 (1.7)
Cardiovascular agents		
Amlodipine besilate	2 (3.2)	1 (1.7)
Olmesartan medoxomil	1 (1.6)	1 (1.7)
Azilsartan	1 (1.6)	0 (0.0)
Diltiazem hydrochloride	1 (1.6)	0 (0.0)
Pitavastatin calcium	2 (3.2)	0 (0.0)
Atorvastatin calcium hydrate	1 (1.6)	0 (0.0)
Lovastatin	1 (1.6)	0 (0.0)
Pravastatin sodium	0 (0.0)	1 (1.7)
Simvastatin	0 (0.0)	1 (1.7)
Immunosuppressive agents, Hormones, and Metabolic agents		
Baricitinib	0 (0.0)	1 (1.7)
Prednisolone	1 (1.6)	2 (3.3)
Insulin human (genetical recombination)	1 (1.6)	1 (1.7)
Potassium citrate and sodium citrate hydrate	2 (3.2)	0 (0.0)
Allopurinol	1 (1.6)	0 (0.0)
Febuxostat	1 (1.6)	0 (0.0)
Colchicine	0 (0.0)	1 (1.7)

Nervous system agents		
Betahistine mesilate	1 (1.6)	0 (0.0)
Pregabalin	0 (0.0)	1 (1.7)
Tramadol hydrochloride and acetaminophen	0 (0.0)	1 (1.7)
Eszopiclone	0 (0.0)	1 (1.7)
Ramelteon	0 (0.0)	1 (1.7)
Zolpidem tartrate	0 (0.0)	1 (1.7)
Respiratory organ agents		
Dextromethorphan hydrobromide hydrate	9 (14.3)	11 (18.3)
Dihydrocodeine phosphate, dl-methylephedrine hydrochloride and Chlorpheniramine maleate	1 (1.6)	0 (0.0)
Dimemorfan phosphate	0 (0.0)	2 (3.3)
dl-Methylephedrine hydrochloride, dihydrocodeine phosphate, diprophylline, diphenhydramine salicylate, acetaminophen and bromovalerylurea	0 (0.0)	1 (1.7)
Tipepidine hibenzate	0 (0.0)	1 (1.7)
Codeine phosphate hydrate	3 (4.8)	2 (3.3)
L-Carbocisteine	4 (6.3)	3 (5.0)
Ambroxol hydrochloride	1 (1.6)	1 (1.7)
Eprazinone hydrochloride	1 (1.6)	0 (0.0)
Ciclesonide	1 (1.6)	0 (0.0)
Budesonide and formoterol fumarate dihydrate	1 (1.6)	0 (0.0)
Mometasone furoate monohydrate	1 (1.6)	0 (0.0)
Fexofenadine Hydrochloride	6 (9.5)	1 (1.7)
Desloratadine	2 (3.2)	0 (0.0)
d-Chlorpheniramine maleate	1 (1.6)	0 (0.0)
Levocetirizine hydrochloride	1 (1.6)	0 (0.0)
Loratadine	1 (1.6)	0 (0.0)
Bilastine	0 (0.0)	1 (1.7)
Olopatadine hydrochloride	0 (0.0)	1 (1.7)

Traditional Chinese medicines		
Anchusan	1 (1.6)	0 (0.0)
Maobushisaishinto	1 (1.6)	0 (0.0)
Shoseiryuto extract	1 (1.6)	0 (0.0)
Hochuekkito extract	0 (0.0)	1 (1.7)
Kakkonto extract	0 (0.0)	1 (1.7)
Kikyoto	0 (0.0)	1 (1.7)

(Appendix)

Collaborators

Nagasaki University Hospital

Clinicians, Investigators, Technicians: Nobuyuki Ashizawa, Takahiro Takazono, Kazuko Yamamoto, Yoshifumi Imamura, Koya Ariyoshi, Hirotomo Yamanashi, Kensuke Takahashi, Kosuke Matsui, Mai Izumida, Takashi Sugimoto, Momoko Yamauchi, Kazuma Iwata, Shogo Akabame, Takeshi Tanaka, Masato Tashiro, Ayumi Fujita, Kosuke Kosai, Kenji Ota, Susumu Fukahori, Atsuko Hara, Shinnosuke Takemoto, Hiroshi Gyotoku, Takayuki Suyama, Takashi Kitamikado, Hideo Yada, Tatsuya Ukawa, Nobuhiro Kanie, Koki Shimizu, Masumi Shimizu, Shuhei Ideguchi, Naoki Iwanaga, Kazuaki Takeda, Takuto Miyamura

Local Clinical Research Team: Mihoko Takada, Tomomi Kobashikawa, Masako Tsurumaru, Yasuko Maeda, Megumi Koga, Seiko Yoshida, Yumi Yamashita, Kazumi Takada, Takako Iwakuma, Midori Watarizaki, Yayoi Nishiuchi, Chizuru Monguchi, Akiko Sumi, Noriko Tanigawa, Kiyomi Matsuo

Chiba University Hospital

ARO Team: Eri Imanishi, Keisuke Jin, Toshinori Nakajima, Yasuhisa Fujii, Kirika Murakami, Mio Ohashi, Michiko Hanawa, Asahi Takahashi, Tadami Fujiwara

National Institute of Infectious Diseases, Department of Fungal Infection

Laboratory Testing Team: Keiko Fukuda, Nobuko Nakayama

National Institute of Infectious Diseases, Department of Bacteriology

Viral sequencing Team: Naoko Ishii, Masatomo Morita

Fukuoka University Chikushi Hospital

Clinician**:** Hisako Kushima

Tokai University, Undergraduate School of Medicine, Faculty of Medicine

Investigator**:** Norio Yamamoto

Osaka City Juso Hospital, Department of Respiratory Medicine

Clinician**:** Satoshi Shiraishi

IMSUT Hospital, The Institute of Medical Science, The University of Tokyo

Clinician**:** Eisuke Adachi, Hiroyuki Nagai,

Clinical Research Center**:** Minako Kono, Saori Minote, Riyo Owada

Saitama Medical Center

Clinicians, Investigators, Technicians**:** Hideaki Oka, Kazuyuki Mimura, Daisuke Ono, Yusuke Nishida, Akio Kanazawa, Yujin Nozaki, Risa Sakai, Takayuki Kawamura, Kei Yamamoto, Hirotaka Yamashita, Ohki Sato, Mayuko Kawamura, Ayako Shirai, Yusuke Ochiai, Hikari Asai

Local Clinical Research Team**:** Masami Kondo, Yuichiro Ohsawa, Ken Komatsuzaki, Reiko Kamimasuda, Risa Komatsu
